# ABCB1 and ABCC1 Function during TGF-β-Induced Epithelial-Mesenchymal Transition: Relationship between Multidrug Resistance and Tumor Progression

**DOI:** 10.3390/ijms24076046

**Published:** 2023-03-23

**Authors:** Kelli Monteiro da Costa, Leonardo Freire-de-Lima, Leonardo Marques da Fonseca, José Osvaldo Previato, Lucia Mendonça-Previato, Raphael do Carmo Valente

**Affiliations:** 1 Laboratório de Glicobiologia, Instituto de Biofísica Carlos Chagas Filho, Universidade Federal do Rio de Janeiro, Rio de Janeiro 21941-902, Brazil; kellimc85@biof.ufrj.br (K.M.d.C.); lfonseca@biof.ufrj.br (L.M.d.F.); previato@biof.ufrj.br (J.O.P.); luciamp@biof.ufrj.br (L.M.-P.); 2 Laboratório de Biologia Celular de Glicoconjugados, Instituto de Biofísica Carlos Chagas Filho, Universidade Federal do Rio de Janeiro, Rio de Janeiro 21941-902, Brazil; leolima@biof.ufrj.br; 3 Núcleo Multidisciplinar de Pesquisa em Biologia (Numpex-Bio), Campus Duque de Caxias Professor Geraldo Cidade, Duque de Caxias, Universidade Federal do Rio de Janeiro, Rio de Janeiro 25250-470, Brazil

**Keywords:** cancer, epithelial-mesenchymal transition, TGF-β, ABC transporters, P-glycoprotein, MRP1, drug resistance, tumor progression

## Abstract

Multidrug resistance (MDR) and induction of metastasis are some of the puzzles encountered during cancer chemotherapy. The MDR phenotype is associated with overexpression of ABC transporters, involved in drug efflux. Metastasis originates from the epithelial-mesenchymal transition (EMT), in which cells acquire a migratory phenotype, invading new tissues. ABC transporters’ role during EMT is still elusive, though cells undergoing EMT exhibit enhanced ABCB1 expression. We demonstrated increased ABCB1 expression but no change in activity after TGF-β-induced EMT in A549 cells. Moreover, ABCB1 inhibition by verapamil increased snail and fibronectin expression, an event associated with upregulation of ABCB1, evidencing coincident cell signaling pathways leading to ABCB1 and EMT-related markers transcription, rather than a direct effect of transport. Additionally, for the first time, increased ABCC1 expression and activity was observed after EMT, and use of ABCC1 inhibitors partially inhibited EMT-marker snail, although increased ABCC1 function translated into collateral sensibility to daunorubicin. More investigations must be done to evaluate the real benefits that the gain of ABC transporters might have on the process of metastasis. Considering ABCC1 is involved in the stress response, affecting intracellular GSH content and drug detoxification, this transporter could be used as a therapeutic target in cancer cells undergoing EMT.

## 1. Introduction

During the 20th century, the world observed a drastic change in the causes of mortality due to the advent of medical-sanitary techniques and the increase in life expectancy. In the early 1900s, the leading cause of death was infectious diseases with a minor proportion of cancer casualties. Nowadays, cancer is one of the main public health problems, being the second cause of deaths worldwide, with approximately 10 million deaths in 2019 [1,2]. Among the types of cancers, lung cancer is the second in terms of the number of new cases (11.4%) and the first in terms of number of deaths (18%), according to data from the International Agency for Research on Cancer [3]. The main risk factors associated lung cancer are related the environment such as tobacco smoking and exposure to asbestos, followed by a history of infectious lung diseases, such as tuberculosis and pneumonia, and non-infectious lung diseases, such as asthma [4].

Cancer is generically defined as a set of diseases resulting from multifactorial processes, in which functional capacities are acquired as cells pass to a malignant neoplastic state [5]. Among the factors, drug resistance is a major obstacle to cancer chemotherapy. Resistance may be intrinsic to the tumor or induced by long-term exposure to drugs. The acquisition of resistance to chemically and functionally unrelated drugs is associated to overexpression of ABC (ATP Binding Cassette) transporters, which basically reduce the drug accumulation in cytosol, reducing in the efficiency of chemotherapy. The multidrug resistance (MDR) phenotype may occur as a result of multifactorial mechanisms, including the increased expression of ABC transporters [6]. These transporters share conserved sequences in the ABC module, using the energy of ATP hydrolysis to transport various molecules across biological membranes against their concentration gradient [7]. ABCB1 (also known as P-glycoprotein/P-gP) and ABCC1 (MRP-1) were the first ABC transporters directly linked to a MDR phenotype [8,9] in cancer cells. However, ABCB1 and ABCC1 transporters are also expressed in normal cells, participating in endo- and xenobiotic excretion, cellular detoxification [10] and stress response [11]. In humans, ABCC1 is ubiquitously expressed, while ABCB1 is expressed in barrier tissues such as the blood–brain barrier, blood–testis barrier, placenta and intestine as well kidney, liver and adrenal gland [10,11,12]. ABCB1 transports a wide variety of molecules, whose chemical characteristics are weakly amphipathic and relatively hydrophobic, generally containing aromatic rings and a positively charged nitrogen atom [13]. On the other hand, ABCC1 transports a wide variety of amphipathic organic compounds containing large, usually negatively charged, hydrophobic groups, either alone or in co-transport or conjugation with glutathione (GSH), sulfate or glucuronide [14].

Several studies have reported a correlation between cancer resistance and increased invasive potential when compared to sensitive cancer cells [15,16]. During the progression of solid tumors, cells undergo a series of complex biological events that result in migration, invasion and colonization of tissues adjacent or anatomically distant from the primary tumor site. The process is collectively named as metastasis, culminating in the spread of cancer and leading to a poor prognosis for chemotherapy efficacy, as well as overall survival [17]. In carcinomas (tumors of epithelial origin), cancer cells undergo a process of transdifferentiation, gaining a migratory and invasive phenotype due to the execution of a multifaceted program known as epithelial-mesenchymal transition (EMT), also observed during embryonic morphogenesis and in tissue regeneration [18]. Basically, cells lose epithelial characteristics with a reduction in the expression of molecules involved in cell-cell adhesion and cell-extracellular matrix as well as of the apical-basal polarity, while concomitantly gaining typical characteristics observed in mesenchymal cells, such as transient adhesion and increased motility [19].

**Figure 1:**
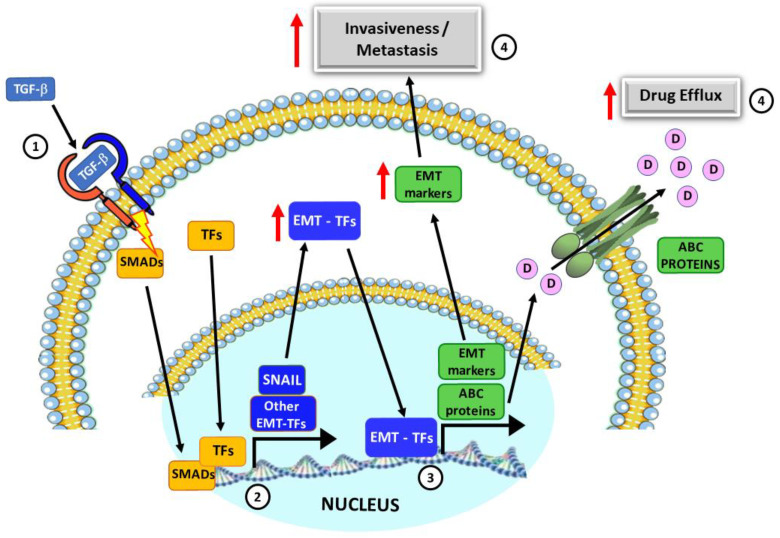
Cell signaling pathways triggered by TGF-β during epithelial mesenchymal transition (EMT) in cancer cells. (1) TGF-β binding to its receptor induces the activation of SMAD proteins in the cytosol, which are translocated into the nucleus together with other transcription factors (TFs), activated by other stimuli. (2) SMADs and other TFs promote the transcription of SNAIL and other EMT-transcription factors (EMT-TFs) that accumulate in the cytosol and are further translocated to the nucleus leading to the transcription of several mesenchymal-specific genes, or EMT markers (e.g., fibronectin and N-cadherin) and ABC transports (ABCB1, ABCC1 and ABCG2). (3) Enhanced expression of EMT markers leads to the onset of a mesenchymal phenotype, which present increased motility and invasiveness, including to distant sites (metastasis). (4) Enhanced ABC protein expression leads to increased extrusion of either drugs or other toxic compounds, leading to multidrug resistance.

The production of the growth factor TGF-β by the tumor microenvironment is one of the components capable of inducing EMT [20] via activation of several transcription factors that orchestrate the process: snail [21], slug [22], zeb1 [23] and twist-1 [24]. The reduction in the expression of E-cadherin, a calcium-dependent cell-cell adhesion protein expressed in epithelial cells, is very well known during EMT, being regulated by the aforementioned transcription factors, especially snail [25,26,27]. In parallel, there is an upregulation of N-cadherin, a mesenchymal protein involved in transient cell-cell adhesion [23]. Additionally, the synthesis and deposition of fibronectin (FN), an extracellular matrix glycoprotein produced by fibroblasts and epithelial cells undergoing EMT, are an important mechanism induced by TGF-β signaling by snail activation to stimulate the invasive phenotype in metastatic niches [18,28]. After EMT, cells need to survive in the peripheral or lymphatic circulation to be able to adhere to new tissue, initiating the mesenchymal-epithelial transition for proliferation and colonization of the metastatic niche [5]. In the process, cells become resistant to apoptosis and, consequently, to chemotherapy, with snail playing an important role, since reducing its expression decreases metastasis and immunosuppression [29] in addition to sensitizing strains resistant to antineoplastic chemotherapeutics such as cisplatin [30,31], paclitaxel [32], 5-fluorouracil and gemcitabine [33] (summarized in Figure 1).

The relationship between ABC proteins and metastasis is still not fully understood. Nonetheless, it has already been observed that the ABCB1 and ABCC1 activities are important for the T cell migration from spleen and peripheral blood to the lymph nodes, in a process driven by the chemokines CCL19 and CCL21 and induced by the immunosuppressant FTY720 [34]. ABCB1 expression was also implicated in enhanced migration and invasion of human trophoblasts in placenta [35]. In the cancer context, Landreville et al. (2011) demonstrated that uveal melanoma patient samples contained a side population (SP) that expressed high levels of ABCB1. ABCB1^+^ melanoma cells exhibited higher ability to form colonies in vitro and to promote both increased tumor growth and number of metastasis in vivo, when compared to ABCB1^−^ cells [36].

With respect to the resistant phenotype and EMT, Li et al. (2009) observed the ABCB1 expression only in breast cancer cells undergoing EMT induced by the chemotherapy adriamycin, with involvement of Twist1 [37]. On the other hand, studies directly evaluating the role of the TGF-β on the ABCB1 function showed conflicting effects, as it was seen that endothelial cells of the cerebral microvasculature suffered an increase in their activity, while gliomas obtained from patients that naturally expressed high levels of ABCB1 suffered inhibition of activity when incubated with cytokine [38,39]. It is noteworthy that ABCC1, ubiquitously expressed in mammals, has not even been mentioned in the literature in this context of cancer progression. Considering the relationship between ABC transporters and cancer progression is not fully established, through the evaluation of TGF-β-induced EMT effects over the expression and function of ABCB1 and ABCC1 transporters in the A549 cell line, we would be able to establish an implication of the MDR phenotype acquisition in metastatic tumors and identify potential therapeutic targets.

## 2. Results

**Figure 2:**
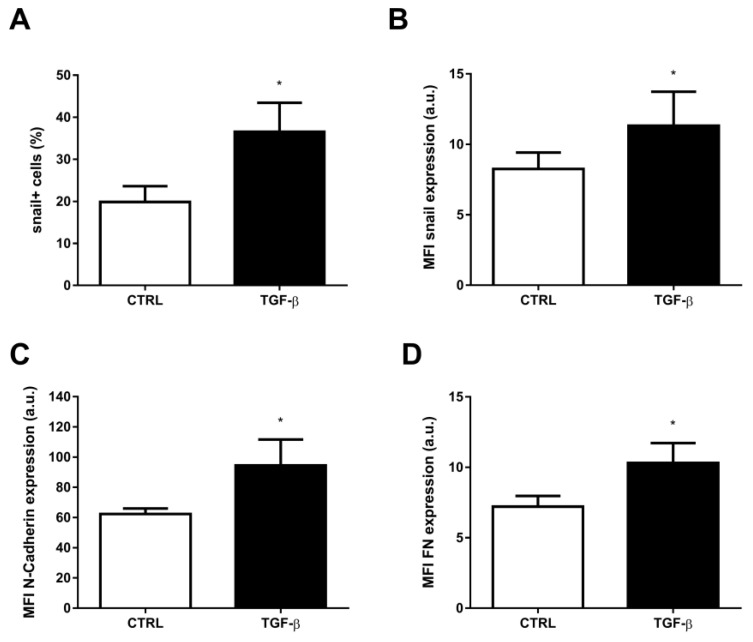
TGF-β increases expression of proteins related to epithelial-mesenchymal transition (EMT). A549 cells (1 × 10^5^ cells/mL) were incubated with 5 ng/mL TGF-β for EMT induction. After 48 h, the cells were stained with antibodies against (**A**,**B**) snail, (**C**) N-cadherin and (**D**) fibronectin (FN) and (**A**) percentage of positive cells and (**B**–**D**) protein expression were evaluated by flow cytometry. Expression is represented by median fluorescence intensities (MFI). Bars represent mean + SEM, and the values of significance were represented by (*) for *p* < 0.05 from at least four independent experiments.

**Figure 3:**
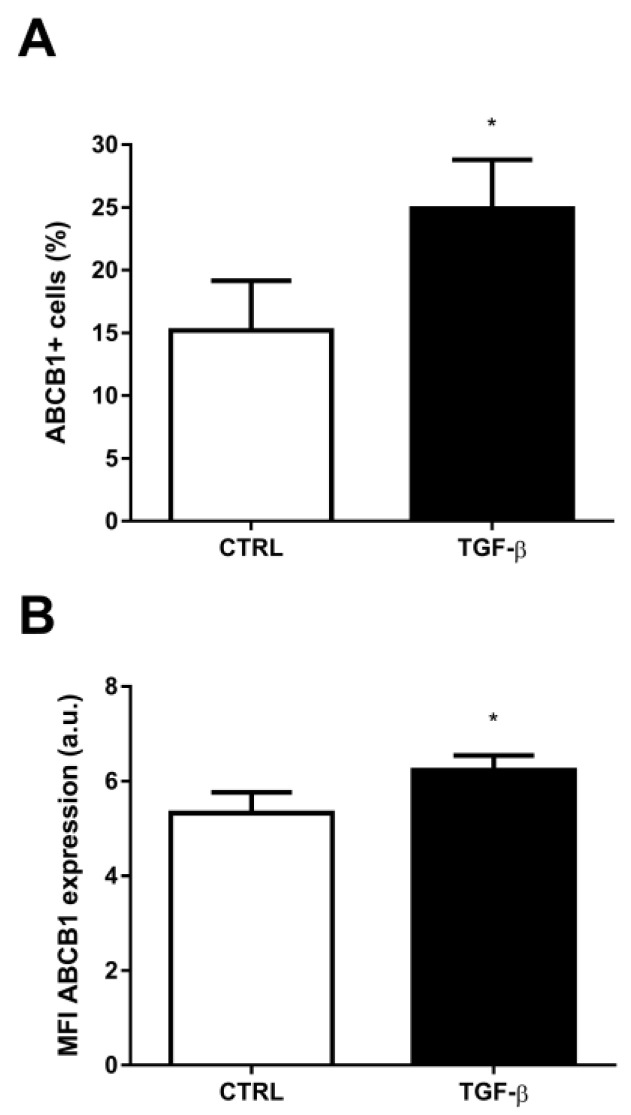
TGF-β increases ABCB1 expression. A549 cells (1 × 10^5^ cells/mL) were incubated with 5 ng/mL TGF-β for EMT induction. After 48 h, the cells were stained with anti-ABCB1 antibody and (**A**) percentage of positive cells and (**B**) protein expression were evaluated by flow cytometry. Expression is represented by median fluorescence intensities (MFI). Bars represent mean + SEM, and the values of significance were represented by (*) for *p* < 0.05 from six independent experiments.

According to previous data, TGF-β is a known inducer of the EMT program [20,40]. Corroborating those findings, in our experimental model, the incubation of A549 cell line for 48 h incubation with this growth factor induced an increase in the percentage of snail^+^ cells, from 19.85 ± 3.78 (mean ± SEM) in the control to 36.54 ± 6, 91 in the presence of TGF-β (Figure 2A). The percentage of cells for N-cadherin^+^ did not change and FN^+^ increased (Supplementary Figure S1). The median fluorescence intensity (MFI) value for snail expression increased by 37.16% (Figure 1B). The results were similar when we evaluated the N-cadherin and FN expression, which had an increase of about 52% and 43%, respectively, under the same conditions (Figure 2C,D). Moreover, incubation with TGF-β was also responsible for the increase in the percentage of cells expressing the ABCB1 efflux transporter, from 15.20 ± 3.96 to 24.89 ± 3.89 (Figure 3A), resulting in an increase of 63.75%. An analysis of the MFI values also revealed an increased ABCB1 expression, with TGF-β-treated cells showing an expression 17.10% higher than control (Figure 3B). Next, we evaluated whether incubation with the cytokine TGF-β would have a direct effect on the efflux capacity of ABCB1. As described in the Materials and Methods section, this ABCB1 efflux was calculated from the ABC efflux assay, from which we obtained the inhibition index. ABCB1 activity was not detected either in the control or in the presence of verapamil and cyclosporine. Also, no change was seen in ABCB1 activity after TGF-β incubation (Supplementary Figure S2).

**Figure 4:**
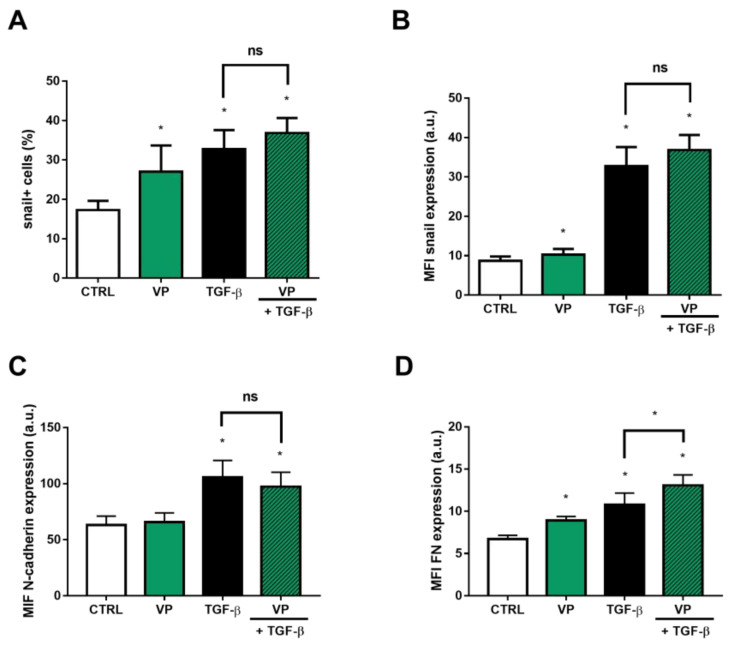
Verapamil changes expression of proteins related to epithelial-mesenchymal transition (EMT). A549 cells (1 × 10^5^ cells/mL) were incubated with 10 µM verapamil for ABCB1 inhibition in the absence or presence of 5 ng/mL TGF-β for EMT induction. After 48 h, the cells were stained with antibodies against (**A**,**B**) snail, (**C**) N-cadherin and (**D**) fibronectin (FN) and (**A**) percentage of positive cells and (**B**–**D**) protein expression were evaluated by flow cytometry. Expression is represented by median fluorescence intensities (MFI). Bars represent mean + SEM, and the values of significance were represented by (*) for *p* < 0.05 from at least four independent experiments. ns = not significant.

Since the expression of the ABCB1 efflux transporter increases during the EMT process, we evaluated whether the inhibition of its transporting activity would interfere with the expression of genes associated with the EMT program. For this, verapamil, a known ABCB1 inhibitor, was incubated for 48 h and expression of snail, N-cadherin and FN was analyzed. Inhibition of ABCB1 efflux during EMT, by means of co-incubation with verapamil and TGF-β did not promote any change in MFI values of any of EMT markers, when compared to control cells incubated solely with TGF-β (Figure 4). On the other hand, the sustained inhibition of ABCB1 by verapamil, per se, resulted in an increase in the percentage of snail^+^ cells by 70%, compared to control (Figure 4A). When evaluating MFI values, our data showed that incubation with verapamil increased the levels referring to the protein expression of snail and FN by 18.34% (Figure 4B) and 32.93% (Figure 4D), respectively. However, no difference was observed regarding N-cadherin adhesion protein expression (Figure 4C).

**Figure 5:**
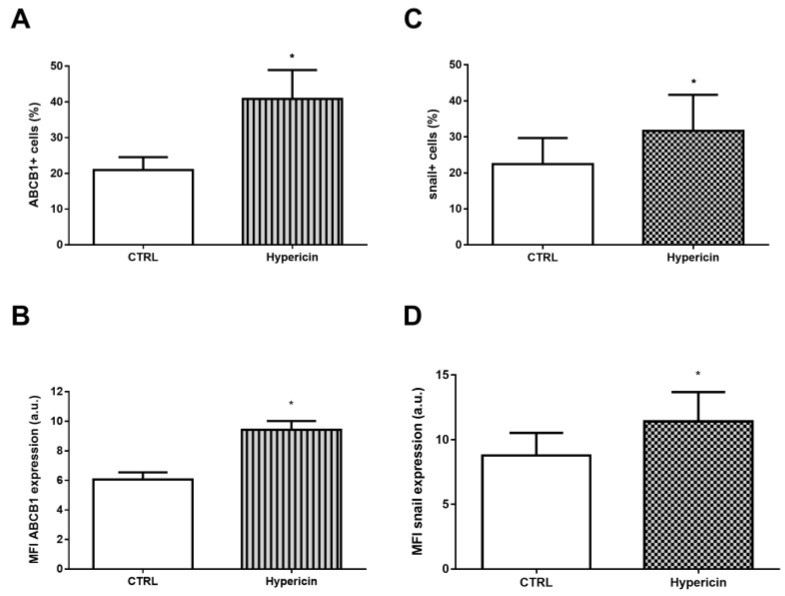
Hypericin increases ABCB1 and snail expression. A549 cells (1 × 10^5^ cells/mL) were incubated with 1.5 µM hypericin for ABCB1 expression induction. After 48 h, the cells were stained with (**A**,**B**) anti-ABCB1 or (**C**,**D**) anti-snail antibodies and (**A**,**C**) percentage of positive cells and (**B**,**D**) protein expression were evaluated by flow cytometry. Expression is represented by median fluorescence intensities (MFI). Bars represent mean + SEM, and the values of significance were represented by (*) for *p* < 0.05 from at least three independent experiments.

Surprisingly, the inhibition of ABCB1 transport increased snail and FN expression. It is noteworthy that some transport inhibitors such as verapamil can also induce increased expression of the transporter [41,42]. In order to analyze whether the observed effect would be related to the efflux inhibition or to the signaling pathways involved in the ABCB1 expression, we induced an increased ABCB1 expression by using hypericin. Hypericin is a molecule of the phenanthropylene quinone class found in plants of the genus *Hypericum* and some types of fungus [43]. In addition to its use in antimicrobial and antitumor photodynamic therapy, the molecule is a well-known inducer of ABCB1 expression [44,45,46]. For this reason, hypericin was added in the absence of TGF-β and ABCB1 and snail expression were analyzed. The 48 h incubation with hypericin induced a 95% increase in the percentage of ABCB1^+^ cells (Figure 5A) and approximately 56% for MFI values (Figure 5B), as described in the literature. The use of hypericin resulted in a 41% increase in the percentage of snail^+^ cells (Figure 5C) and a 30% increase in MFI values for snail (Figure 5D) compared to control cells, suggesting a cross-signaling pathway between these genes.

**Figure 6:**
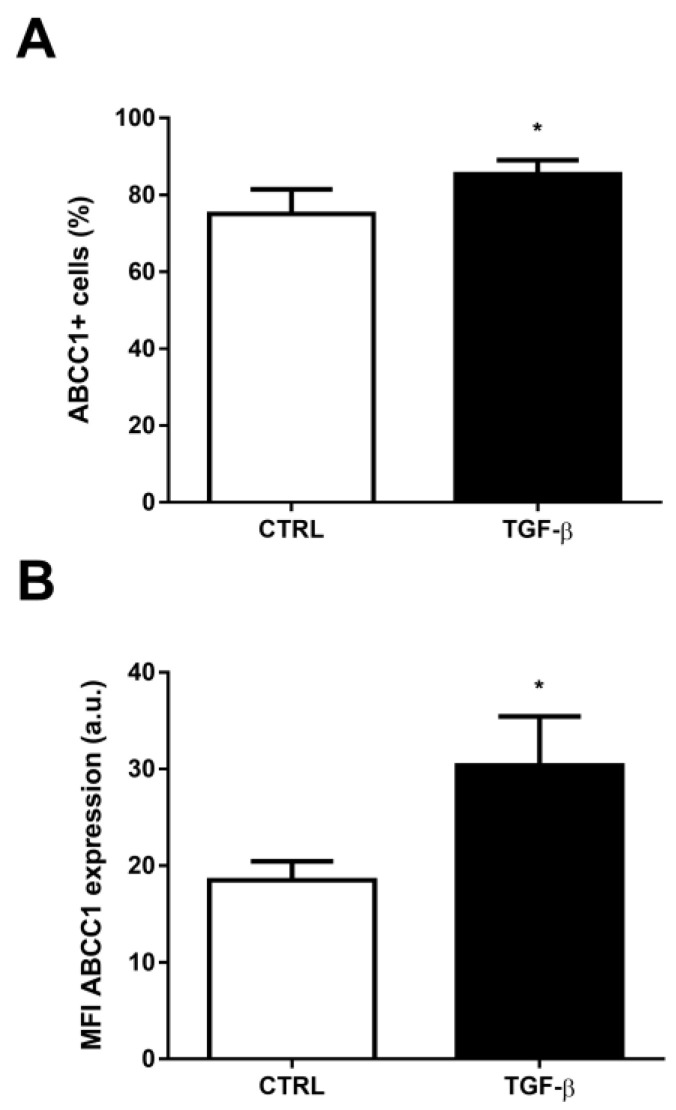
TGF-β-induced epithelial-mesenchymal transition (EMT) increases ABCC1 expression. A549 cells (1 × 10^5^ cells/mL) were incubated with 5 ng/mL TGF-β for EMT induction. After 48 h, the cells were stained with anti-ABCC1 antibody and (**A**) percentage of positive cells and (**B**) protein expression were evaluated by flow cytometry. Expression is represented by median fluorescence intensities (MFI). Bars represent mean + SEM, and the values of significance were represented by (*) for *p* < 0.05 from four independent experiments.

ABCC1 is another ABC efflux transporter involved in cellular detoxification and the cellular stress response, and is important to the MDR phenotype. Despite its ubiquitous expression, no attention has been given to this transporter in the context of the EMT program in cancer. Interestingly, incubation with TGF-β also promoted an increase in the percentage of ABCC1^+^ cells from 75.04 ± 6.37 to 85.32 ± 3.67 (Figure 6A) and in ABCC1 expression from 18.50 ± 1.98 to 30.35 ± 5.09 (Figure 6B). Similarly to ABCB1, TGF-β incubation results an increase of about 14% in the percentage of positive cells and of 64% in transporter expression.

**Figure 7:**
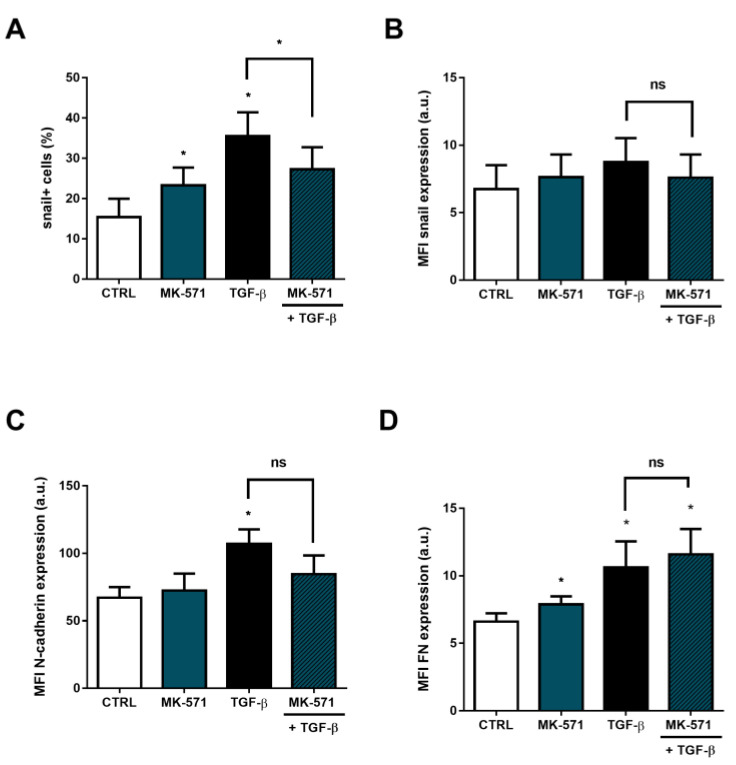
MK-571 changes the expression of epithelial-mesenchymal transition (EMT)-related proteins. A549 cells (1 × 10^5^ cells/mL) were incubated with 25 µM MK-571 for ABCC1 inhibition in the absence or presence of 5 ng/mL TGF-β for EMT induction. After 48 h, the cells were stained with antibodies against (**A**,**B**) snail, (**C**) N-cadherin and (**D**) fibronectin (FN) and (**A**) percentage of positive cells and (**B**–**D**) protein expression were evaluated by flow cytometry. Expression is represented by median fluorescence intensities (MFI). Bars represent mean + SEM, and the values of significance were represented by (*) for *p* < 0.05 from at least three independent experiments. ns = not significant.

Therefore, we analyzed whether inhibition of ABCC1 efflux could also interfere with the expression of snail, N-cadherin and FN. For this purpose, the cells were incubated with MK-571, a specific ABCC1 inhibitor, for 48 h in the presence and absence of TGF-β. The results in the absence of TGF-β were similar to those found for ABCB1: snail and FN levels showed some elevation in the presence of the inhibitor in relation to untreated cells, in which MK-571 remarkably increased the percentage of snail+ cells by 51.03% and FN expression levels by 19.39%, when compared to control cells (Figure 7A,D). This increase in the percentage of snail+ cells did not translate into changes in MFI values for snail, which was similar to those of control cells (Figure 7B). Likewise, N-cadherin showed no change in MFI values either (Figure 7C). However, when considering the effects of ABCC1 inhibition during EMT, our data showed that incubation of the inhibitor with TGF-β reduced the percentage of snail+ cells by 23.19% in relation to cells treated only with TGF-β (Figure 6A). No significant effects were detected in either N-cadherin or FN levels, when compared to cytokine-treated cells (Figure 7C,D), an indication that ABCC1 function may only partially affect EMT induction.

**Figure 8:**
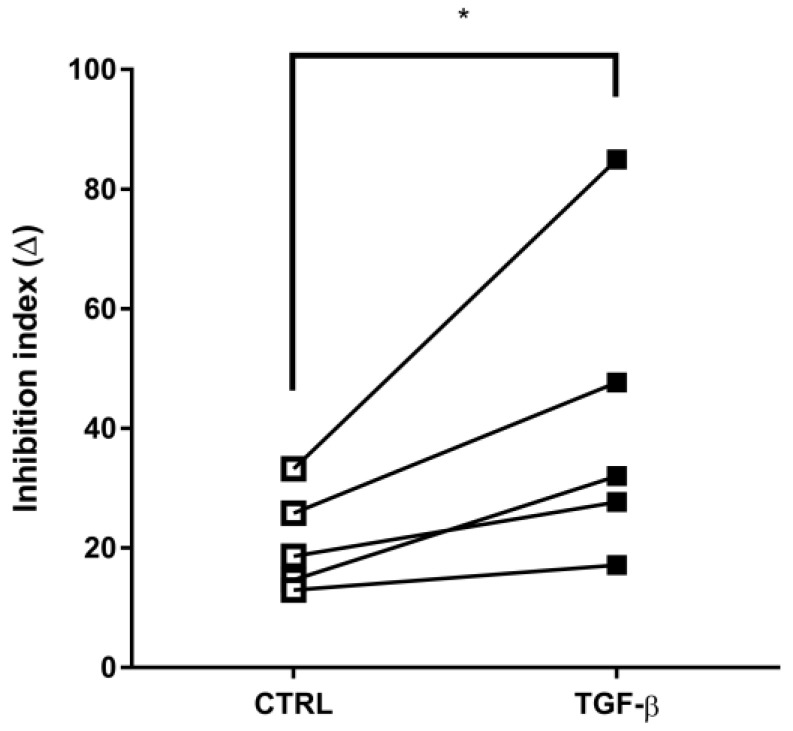
TGF-β-induced epithelial-mesenchymal transition (EMT) enhances ABCC1 activity. A549 cells (1 × 10^5^ cells/mL) were incubated with 5 ng/mL TGF-β for EMT induction. After 48 h, ABCC1-mediated efflux assays were performed as described in Material and Methods. ABCC1 activity is represented by inhibition index (Δ). Squares and lines represent each paired experiment. Values of significance were represented by (*) for *p* < 0.05 from five independent experiments.

Taking into account the striking induction of ABCC1 protein expression, we next decided to evaluate whether its transport activity would be modified during EMT. Unlike what was observed for ABCB1, incubation with TGF-β significantly increased ABCC1 activity. The inhibition index for this transporter increased from 21.07 + 3.75 in the control to 41.93 + 11.93 in TGF-β treated cells, representing an increase of almost 100% (Figure 8).

**Figure 9:**
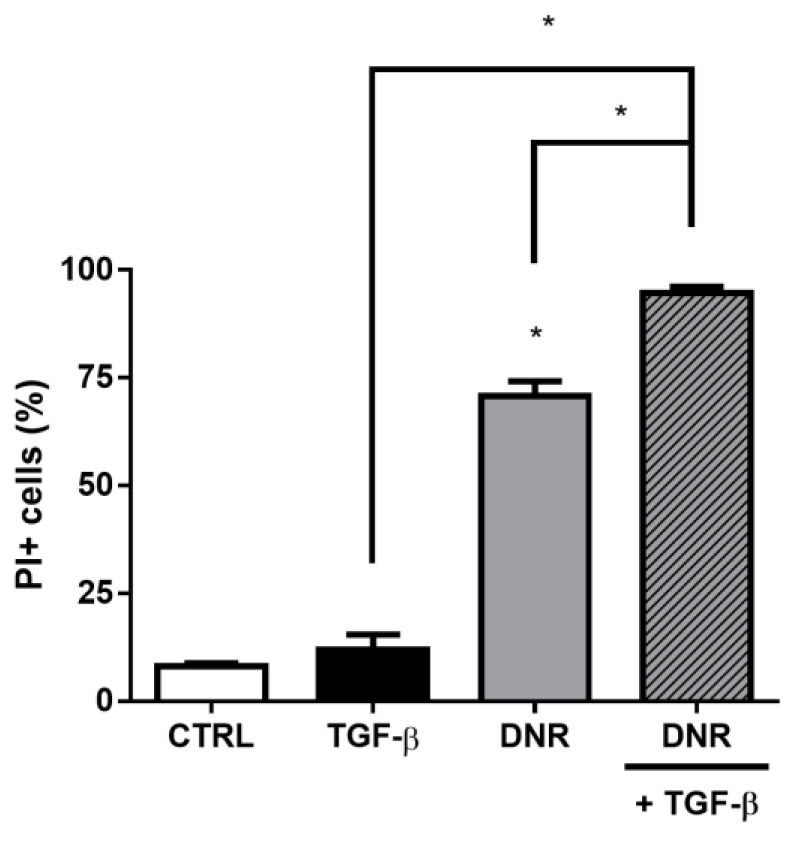
Daunorubincin-induced cytotoxicity increases during epithelial-mesenchymal transition (EMT). A549 cells (1 × 10^5^ cells/mL) were incubated with 5 ng/mL TGF-β for EMT induction. After 48 h, cells were incubated with 500 nM daunorubicin for death induction. After, 24 h, cells were stained with propidium iodide (PI) and percentage of positive cells (non-viable cells) were evaluated by flow cytometry. Bars represent mean + SEM, and the values of significance were represented by (*) for *p* < 0.05 from four independent experiments.

Increased expression of ABC transporters is related to the acquisition of the resistant phenotype. Since TGF-β increases the expression of ABCB1 and ABCC1, mainly increasing ABCC1 activity, we evaluated whether cells treated with TGF-β would be resistant to the induction of cell death by daunorubicin. This drug is an anthracycline with antibiotic and antitumor properties, which induces DNA damage mediated by quinone-generated redox activity, intercalation distortion of the DNA double helix or stabilization of the cleavable complex formed between DNA and topoisomerase II [47]. Additionally, daunorubicin is transported by ABCB1 [13] and by ABCC1 [48]. According to the literature, treatment with daunorubicin increases the percentage of PI^+^ cells, that is, non-viable cells, from 8.04 ± 0.73 to 70.67 ± 3.52 (Figure 9). As expected, the cytokine TGF-β showed no toxic effect on A549 cells. Unlike what was expected, cells pretreated with TGF-β did not show resistance to daunorubicin. After 48 h of incubation with TGF-β for EMT induction and increase of ABC transporters, the addition of daunorubicin increased the percentage of PI^+^ cells to 94.70 ± 1.43 in 24 h, suggestive of the collateral sensitivity phenomenon.

## 3. Discussion

Regarding cancer treatment, resistance to chemotherapy still figures as a major concern to clinicians, as many tumors may acquire the MDR phenotype after exposure to chemotherapy drugs. Among several characteristics related to the induction of MDR phenotype, the overexpression of ABC transporters such as ABCB1 and ABCC1 is a remarkable indicative of poor prognosis [49,50,51,52,53]. Moreover, it has been postulated that tumor resistance may be a result of cancer progression, as several studies have described a relationship between EMT induction and increased resistance to treatment.

In 2007, Cheng and co-workers demonstrated that Twist-1 activates AKT2 in breast cancer cells inducing increased invasion, migration and improved resistance to paclitaxel [54]. More recently, it has been shown that overexpression of EMT transcription factors slug and snail in lung adenocarcinoma cell lines promoted resistance to the inhibitor of tyrosine kinases gefitinib. On the other hand, gefitinib-resistant cell lines maintained in long-term cultures in gefitinib-free medium exhibited regained sensitivity to the treatment with tyrosine kinase inhibitors, phenomena correlated with low expressions of both snail and slug, and also vimentin, thus demonstrating a reversion of the EMT phenotype [55].

Corroborating those findings, Wu and co-workers (2019) demonstrated in lung cancer cell lines (A549 and SPC-A-1) that the overexpression of the transcription factor regulator PAX-6 was correlated with enhanced expression of ZEB2 transcription factor, one of the master regulators of EMT, and also that the ZEB2 promoter region was a direct target for interaction with PAX-6. Additionally, PAX-6 overexpression induced increased migration and invasion in in vitro and in vivo models, and PAX-6 overexpressing cells concomitantly exhibited high resistance against cisplatin toxicity. Conversely, silencing of PAX-6 by SiRNA reverted all those mentioned processes [56].

The upregulation of ABCB1 following EMT induction was demonstrated in vitro in experiments using adriamycin, in which cells undergoing EMT, assessed via twist-1 upregulation, exhibited an increase of ABCB1 expression. However, such an event was also correlated with significant induction of cell death [37]. Recently, our group demonstrated that adenocarcinoma cells resistant to cisplatin indeed displayed increased expression of the EMT markers N-cadherin and FN and decreased expression of the epithelial marker E-cadherin. Moreover, those cisplatin-resistant cells exhibited higher migration than parental cells and increased expression of ABC transporters ABCB1, ABCC1 and ABCG2 [57].

It is remarkable that most of the studies evaluating the relationship between ABC proteins and cancer progression focused on the effects of cancer drugs. Additionally, they emphasized solely the expression of ABC proteins and did not assess the effects of EMT on the transport activity of ABC proteins. Thus, in the present work, we aimed to assess the role of ABC transporters during EMT induced by physiological mediator TGF-β. Corroborating previous results, in our model, TGF-β-induced EMT was assessed by means of increased expression of mesenchymal markers FN and N-cadherin, and also induction of the EMT transcription factor snail. In addition, EMT induction was correlated with a slight increase of ABCB1 expression, which was detected only in a small fraction of A549 cell population. Conversely, physiological EMT induced a notable upregulation of ABCC1 transporter.

As mentioned before, several studies have pointed to a link between signaling pathways involved in upregulation of both EMT transcription factors and ABC transporters [54,55,56,57], but there were no data available concerning the role of transporting activity of ABC proteins during EMT cell programming was available so far. In this context, we performed inhibition of either ABCB1 or ABCC1 efflux during EMT induction to evaluate a direct role of ABC transporters on EMT cellular changes. Evaluating ABCB1 blockage during EMT, it was observed that inhibition with verapamil induced a slight increase in FN expression. We cannot conclude that such an event was indeed related to an increase in EMT pathways, since no alteration in both snail and N-cadherin expression was detected when comparing TGF-β alone and TGF-β plus verapamil. On the other hand, we observed that solely the sustained inhibition of ABCB1 for 48 h with verapamil induced a remarkable increase in snail-positive cells and in FN expression.

Considering it has been long known that sustained pharmacological inhibition of ABCB1 may lead to an increase on its own expression [58] and that verapamil acts as a competitive inhibitor of this transporter, we aimed to evaluate whether snail upregulation was correlated to the impairment of ABCB1 activity or, in turn, could be a result of cellular signaling pathways that could confer both upregulation of ABCB1 and snail. In order to address that matter, we made use of hypericin, a metabolite synthesized by plants of the genus *Hypericum*, especially *Hypericum perforatum* L. (Saint John’s wort), and a well-known inducer of ABCB1 that do not act as a pharmacological inhibitor [44]. Confirming previous data, hypericin not only induced a significant upregulation of ABCB1 in our experimental model but also promoted an upregulation in snail. Taking into account that hypericin does not alter ABCB1 efflux activity, this suggests that upregulation of both snail and FN after long-term incubation with verapamil was a result of ABCB1 transcription induction, whose pathways exhibit a crosslink with promoters of EMT transcription factors, rather than pointing to a direct role of ABCB1 transport impairment on EMT cellular programming.

Considering ABCB1 relevance during tumor progression, in contrast to previous findings correlating ABCB1 upregulation with enhanced resistance to chemotherapy [59,60,61], we did not observe any alteration on its activity after TGF-β-induced EMT (Supplementary Figure S2). Adding to the fact that TGF-β incubation only marginally increased ABCB1 levels in a small fraction of the cell population, our results suggest that during mild and endogenous stimuli of tumor progression in A549 cells, ABCB1 may not confer a significant increase of tumor resistance.

Since ABCC1 is ubiquitously expressed [62], and is upregulated in tumor cells under treatment with several chemotherapy drugs, such as doxorubicin and vinblastine [63,64], another aim of the present work was to assess whether this transporter could be implicated in drug resistance following a physiological inducer of tumor progression. Our results demonstrated a significant increment of ABCC1 protein expression during EMT, confirming previous data demonstrating an induction of ABCC1 mRNA levels after TGF-β treatment in breast cancer cells [65]. Nevertheless, once again, as no data focusing on ABCC1 during EMT were available so far, we also addressed whether ABCC1 activity would be relevant to the EMT cellular altered by TGF-β stimulus. Strikingly, we observed, for the first time, that inhibition of ABCC1 activity during EMT partially reverted the induction of snail. Considering ABCC1 activity also regulates intracellular redox status, by extruding either reduced or oxidized GSH [66], and that reactive oxygen species (ROS) participate in the induction of EMT by activation of snail signaling pathway [67], it is possible that ABCC1 inhibition prevented A549 cells to fully undergo EMT, though we cannot exclude that other signaling pathways could be altered when the transporter is fully inhibited.

Taking into account that ABCC1 function seems to be important to the development of EMT, we evaluated whether the activity of this transporter would be altered by TGF-β-induced EMT. Notably, we also observed, for the first time, a significant increase in ABCC1 activity following EMT, denoting a possible practical gain in the ability of extruding chemotherapy drugs or other substrates by tumor cells undergoing this process. In order to verify whether ABCC1 induction during EMT would indeed confer enhanced resistance against chemotherapy, we evaluated a cellular viability assay after incubation with daunorubicin. Strikingly, the induction of cell death was higher in cells after EMT. Although at first glance that result seems conflicting, it has been long known that exacerbated increase in ABCC1 activity may induce higher sensitivity to deleterious actions of ROS and other cell stressors (e.g., drugs or xenobiotics), rather than protecting cells against toxicity [66]. This feature, observed in ABCC1-overexpressing cells, is caused by the depletion of the cellular antioxidant GSH under stress, by means of the increased efflux of reduced (GSH) or oxidized GSH (GSSG), either alone or in conjugation or cotransport with drugs. Considering GSH is the cell’s main non-protein thiol, participating in the cellular stress response by reducing drugs and/or free radicals, rapid efflux of either GSH and GSSG by ABCC1 interferes with the GSH/GSSG balance and, consequently, with the lack of intracellular substrate for the rapid recycling GSH by GSH reductase, making cells more susceptible to oxidative stress [68,69]. For that reason, the cell population with more expression of ABCC1 (associated with the MDR phenotype, and therefore, it is more resistant) is more sensitive to DNR than the parental population, an effect that was named collateral sensitivity and can be ultimately considered the Achilles’ heel of tumor cells overexpressing ABCC1, owing to the increased efflux of both GSH and GSSG in both basal and cytotoxic conditions, making resistant cells more susceptible to pro-oxidant attacks than sensitive cells, which can be used as an antitumor strategy [69].

Several studies have suggested that EMT triggering would confer a stem cell phenotype to cancer cells (cancer stem cells, CSC), with the onset of SP features, such as expression of dedifferentiation markers such as Oct-4 and Nanog, and overexpression of the ABC transporters ABCB1 or ABCG2 [70,71], as reviewed by Mohan and co-workers [72]. The main features of CSCs are very similar to those of hematopoietic stem cells, especially with regard to their self-renewal capacity and drug resistance, which can be easily detected by the SP assay, an evaluation largely employed for the identification of SP in hematopoietic cells, based on the extrusion of distinct dyes by means of increased activity of either ABCB1 or ABCG2 [73]. In the context of cancer, CSCs are known to drive tumor initiation and induce tumor relapse after chemotherapy, posing a significant challenge in cancer treatment. However, our results do not suggest a direct induction of a typical stem cell SP phenotype in A549 cells treated with TGF-β, since we could not observe any increment of ABCB1 activity and also because the upregulation of both ABCC1 expression and activity is not a common feature of hematopoietic SP stem cells.

Nevertheless, it is important to mention that not all SP cells are necessarily stem cells [74] and that distinct subpopulations of SP cells may express particular sets of phenotypic markers, thus belonging to distinct stages of differentiation [75,76]. In that context, it has been described that ABCC1 may be upregulated in both physiological and tumor microenvironments during the acquisition of an SP phenotype. Adipose-derived stromal cells (ASCs) are known to possess a subpopulation of progenitor cells that can give rise to myogenic, osteogenic, chondrogenic and adipogenic lineages [77]. Using immunophenotypic analysis, Andersen and co-workers (2008) have demonstrated that progenitor cells from ASCs exhibits SP phenotype and are a heterogeneous population that can be divided in CD45^+^ and CD45^−^ (from hematopoietic or stromal origin, respectively). Surprisingly, ABCB1 was mainly expressed by cells in the CD45+ subpopulation, while ABCC1 was expressed in both subpopulations [75]. Corroborating these data, it has been recently described that co-culture with mesenchymal stromal cells from bone marrow induced increased activity of ABCB1, ABCG2 and ABCC1 with the development of an SP phenotype from blasts of acute myeloid leukemias obtained from human patients. Although there was an increase in ABCC1 expression and activity in all patients, the degree of modulation varied in each patient [78].

Thus, the results obtained in the present work suggest that the remarkable ABCC1 upregulation was induced by a physiological EMT modulator in A549 cells, probably by the crossover of TGF-β stimulus and ABCC1 transporter signaling pathways. Increased ABCC1 expression disrupts the GSH/GSSG balance and causes collateral sensitivity to DNR. More investigations must be undertaken in order to evaluate the real benefits that the gained from the upregulation of ABCB1 and ABCC1 on invasiveness and aggressiveness of metastatic tumors. Nonetheless, our data propose that ABCC1 protein could also be used as a target for medical interventions in these tumors by exploring their collateral sensitivity by free radical-inducing drugs.

## 4. Materials and Methods

### 4.1. Cell Subculture

The A549 lung adenocarcinoma cell line obtained from the company ATCC (Manassas, VA, USA) was maintained in RPMI 1640 medium (Thermo Fisher Scientific, Waltham, MA, USA) supplemented with 25 mM HEPES (Merk, Burlington, MA, USA), 2 g/L sodium bicarbonate (Merk), 10% fetal bovine serum (SBF, Thermo Fisher Scientific) and 50 µg/mL gentamicin (Merk) at 37 °C in a 5% CO_2_ atmosphere. At 80–90% culture confluency, cells are washed 2× with PBS buffer and untied with 0.25% trypsin solution (Thermo Fisher Scientific) and 0.2 g/L EDTA at 37 °C for 1–2 min. Then, the cells were centrifuged at 200× *g* for 5 min and counted in 0.04% Trypan blue solution (Merk) in a Neubauer chamber. Lastly, 1 × 10^4^ cells/mL are added to a 75 cm^2^ culture flask with 10 mL of fresh complete medium.

### 4.2. Induction of Epithelial-Mesenchymal Transition

In this study, 1 × 10^5^ cells/mL were incubated in complete RPMI medium at 37 °C with 5% CO_2_ in a six-well plate for 24 h for adhesion. Next, the culture medium was replaced by fresh medium containing 5 ng/mL TGF-β (RD systems, Minneapolis, MN, USA). After 48 h, cells were washed 2× with PBS buffer, untied and transferred to 96-well microtiter plate for antibody labeling. For inhibition of ABCB1 and ABCC1 transporters, 10 µM verapamil or 25 µM MK-571 (all from Merk) was added to the culture along with TGF-β for 48 h.

### 4.3. Induction of ABCB1 Transporter Expression

In this study, 1 × 10^5^ cells/mL were incubated in complete RPMI medium at 37 °C with 5% CO_2_ in a six-well plate for 24 h for adhesion. Next, the culture medium was replaced by fresh medium containing 1.5 µM hypericin (Merk). After 48 h, cells were washed 2× with PBS, untied and transferred to 96-well microtiter plate for antibody labeling.

### 4.4. Antibody Labeling

In this study, 1 × 10^5^ cells were centrifuged and suspended in 100 µL of FACS Lysing solution (BD Biosciences, San Jose, CA, USA), diluted in distilled water for 10 min at room temperature for permeabilization and fixation. Then, cells were blocked for non-specific labeling with PBS containing 5% FBS for 30 min at 4 °C. Then, the cells were centrifuged and suspended in PBS containing antibodies mouse anti-human snail (clone: G-7), N-cadherin (clone: H-63), FN (clone: EP5), ABCB1 (clone: D-11) or ABCC1 (clone: QCRL-1) diluted according to the manufacturer Santa Cruz Biotechnology (Dallas, TX, USA) and incubated for 30 min at 4 °C in the dark. Again, the cells were centrifuged and suspended in PBS containing antibody goat anti-mouse IgG coupled to the Alexa Fluor 488 fluorophore (Life Technologies, Eugene, OR, USA), diluted according to the manufacturer. Finally, cells were centrifuged and suspended in PBS containing 5% FBS and kept on ice for acquisition by flow cytometry.

### 4.5. ABCB1 and ABCC1-Mediated Efflux Assay

The efflux assay was divided into 30 min steps: accumulation and efflux of fluorescent substrate. The naturally fluorescent substrate Rho 123 was employed to analyze the ABCB1-mediated efflux assay. Briefly, 1 × 10^5^ cells were washed 2× with PBS and incubated with 250 nM Rho 123 (Merk) diluted in RPMI medium in absence or presence of 1 μM cyclosporin A and 10 μM verapamil, known ABCB1 inhibitors. After the accumulation step, supernatants were discarded and cells were washed 2× with PBS and then suspended in RPMI medium in the absence or presence of the inhibitors for the efflux step. Afterward, supernatants were discarded and cells were washed and suspended in PBS supplemented with 5% FBS and kept on ice for immediate acquisition by flow cytometry. For the ABCC1-mediated efflux assay, the CFDA dye (Life Technologies of Brazil, São Paulo, SP, Brazil) was employed. In the cytosol, CFDA is hydrolyzed into the fluorescent substrate CF, which is transported to extracellular medium by ABCC1. The cells were incubated with 500 nM CFDA diluted in RPMI medium in the absence or presence of 25 µM MK-571, a known ABCC1 inhibitor, for accumulation and efflux steps.

As a negative control, cells were not exposed to dyes. MDR cells Lucena-1 were employed as positive control due to the overexpression of ABCB1 and ABCC1 and donated by Dr. Vivian Rumjanek from the Institute of Medical Biochemistry Leopoldo de Meis, Federal University of Rio de Janeiro, Rio de Janeiro, Brazil.

### 4.6. Assessment of Cellular Viability

The staining of non-viable cells was performed by the DNA intercalation with dye propidium iodide (PI), excluded by viable cells with intact membranes. Here, 1 × 10^5^ cells/mL were incubated in complete RPMI medium at 37 °C with 5% CO_2_ in a six-well plate for 24 h for adhesion. Next, the culture medium was replaced by fresh medium containing 5 ng/mL of TGF-β. After 48 h, 500 nM of daunorubicin was added to the medium and cells were incubated for another 24 h. The cells were harvested, centrifuged and while the supernatants were discarded, the cells suspended in 1 µg/mL PI diluted in PBS and incubated for 15 min prior to acquisition by flow cytometry. As the positive control of cell death, cells were incubated with 4% paraformaldehyde for 10 min before the addition of dye. As autofluorescence control, parasites were not exposed to neither dye nor paraformaldehyde.

### 4.7. Flow Cytometry Analysis

Fluorescence intensities of Alexa-488 fluorophore were acquired on the FL1-H channel (530/30 bandpass filter), while PI dye fluorescence were acquired on the FL3-H channel (670LP filter) of a FACSCalibur (BD Biosciences). Post-analysis was performed in the Summit software (version 4.3, Dako Colorado, Fort Collins, CO, USA) on at least 10,000 viable cells that were gated in accordance to forward (FSC) and side scatter (SSC) parameters representative of cell size and granularity. Median fluorescence intensity (MFI) data of antibody labeling are representative of expression of proteins. For the ABC-mediated efflux assay, MFI and percentages of positive cells were acquired from histograms of fluorescent substrates. A negative/low fluorescence gate was designed containing 95% of control cells from the histogram origin, while a high fluorescence gate contained the remaining in the efflux assay. The inhibition index was calculated as the ratio of MFI in the presence of inhibitor to the one in the absence (Control), in which values under or equal 1 represent no efflux and values higher than 1 indicate the level of ABC activity.

### 4.8. Statistical Analysis

Statistical analysis was performed using the software GraphPad Prism (GraphPad Software Inc., San Diego, CA, USA). The student’s *t*-test was employed for two paired comparisons, while one-way ANOVA tests were employed for more than two comparisons, in parametric populations. Significant values were represented by (*) for *p* < 0.05.

## Data Availability

All the data are contained in the article.

## Supplementary Materials

The supporting information can be downloaded at: https://www.mdpi.com/article/10.3390/ijms24076046/s1.

## Sample Availability

Samples of the compounds are available commercially.

## Abbreviations

ABC	ATP-binding cassette
ABCB1	subfamily B, member 1
ABCC1	subfamily C, member 1
CSC	cancer stem cells
EMT	epithelial-mesenchymal transition
FN	fibronectin
GSH	reduced glutathione
GSSG	oxidized glutathione
MDR	multidrug resistance
MFI	median fluorescence intensities
ROS	reactive oxygen species
SP	side population
