# Supplementary material for: ABCB1 and ABCC1 Function during TGF-β-Induced Epithelial-Mesenchymal Transition: Relationship between Multidrug Resistance and Tumor Progression
Source: Int J Mol Sci. 2023 Mar 23;24(7):6046. doi: 10.3390/ijms24076046 (PMC10093952; doi:10.3390/ijms24076046)
Supplement: Supplementary file 1 [file ijms-24-06046-s001.zip › ijms-2254892-supplementary.pdf]

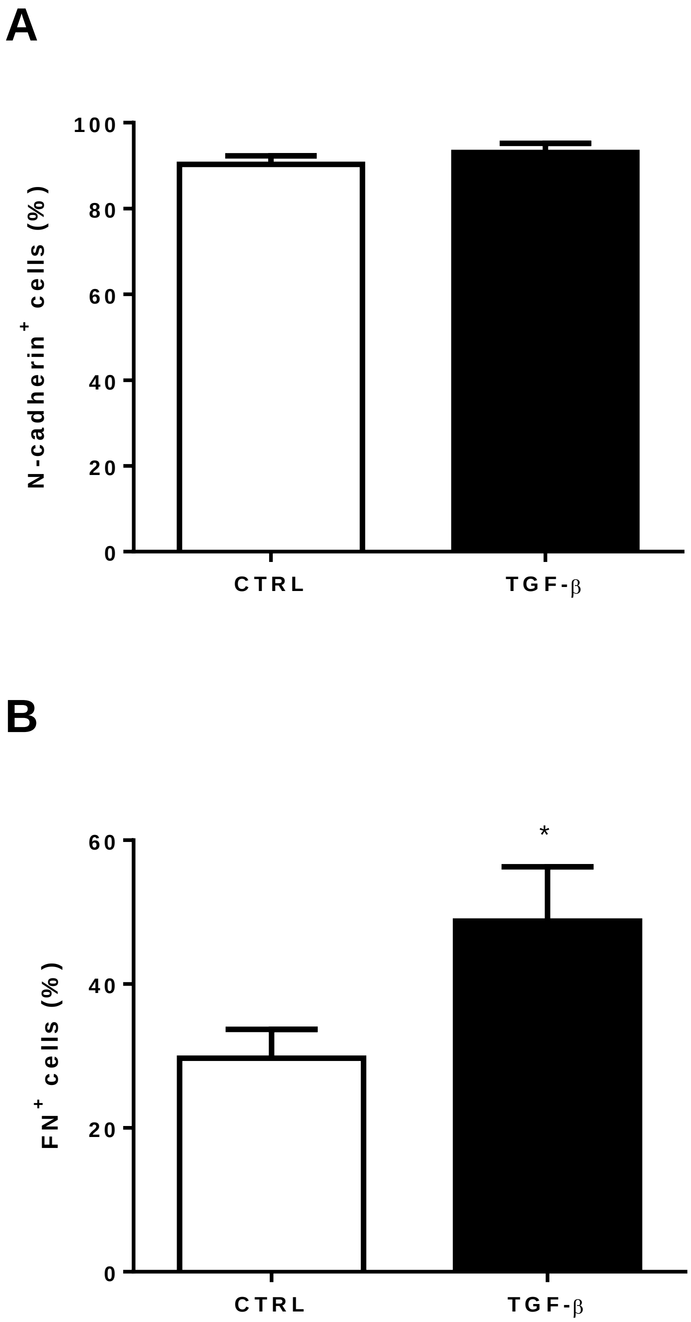


**Supplementary Figure S1.** Percentage of positive cells for N-cadherin and fibronectin (FN) after TGF-β treatment. A549 cells (1 × $10^5$ cells/mL) were incubated with 5 ng/mL TGF-β for epithelial-mesenchymal transition induction. After 48 h, the cells were stained with antibodies against (A) N-cadherin and (B) FN and evaluated by flow cytometry. Bars represent mean + SEM, and the values of significance were represented by (*) for $p < 0.05$ from at least four independent experiments.

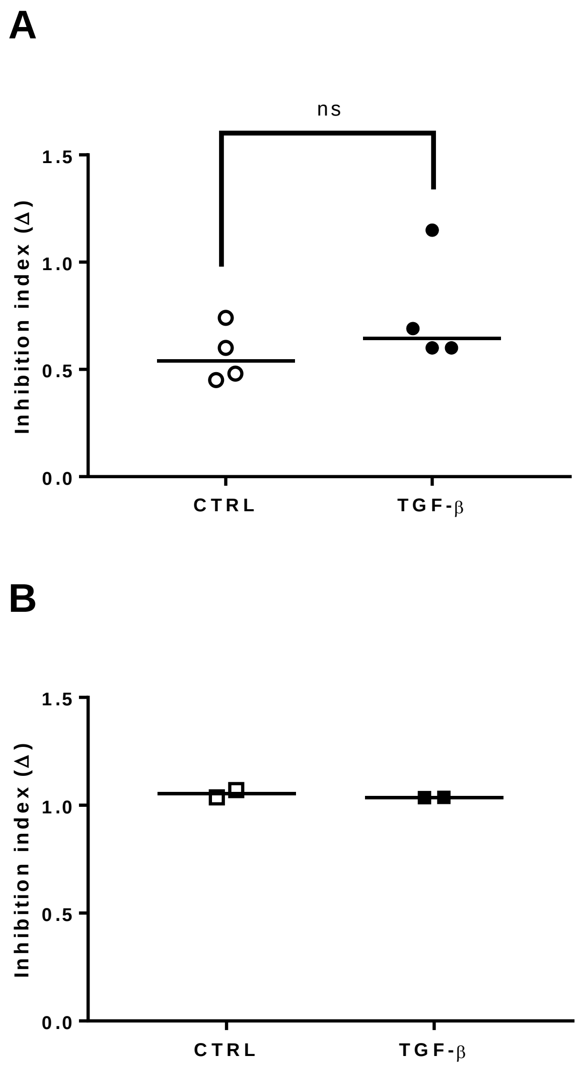


**Supplementary Figure S2.** ABCC1 activity after TGF-β treatment. A549 cells ($1 \times 10^5$ cells/mL) were incubated with 5 ng/mL TGF-β for EMT induction. After 48 h, ABCB1-mediated efflux assays were performed as described in Material and Methods. ABCB1 activity is represented by inhibition index (Δ). ABCB1 activity in the presence of (A) 10 µM verapamil and (B) 1 µM cyclosporin A. ns = not significant. (A) n = 4 and (B) n = 2 independent experiments. The inhibition index was calculated as the ratio of MFI in the presence of inhibitor to the one in the absence (Control), in which values under or equal 1 represent no efflux and values higher than 1 indicate the level of ABCB1 activity.
